# Synthesis of Peptide Nucleic Acids Containing a Crosslinking Agent and Evaluation of Their Reactivities

**DOI:** 10.3390/molecules20034708

**Published:** 2015-03-13

**Authors:** Takuya Akisawa, Yuki Ishizawa, Fumi Nagatsugi

**Affiliations:** Institute of Multidisciplinary Research for Advanced Materials, Tohoku University, 2-1-1 Katahira, Aoba-ku, Sendai-shi, Miyagi 980-8577, Japan; E-Mails: akisawat@mail.tagen.tohoku.ac.jp (T.A.); yi87653@yahoo.co.jp (Y.I.)

**Keywords:** peptide nucleic acid (PNA), crosslink, antisense, invasion

## Abstract

Peptide nucleic acids (PNAs) are structural mimics of nucleic acids that form stable hybrids with DNA and RNA. In addition, PNAs can invade double-stranded DNA. Due to these characteristics, PNAs are widely used as biochemical tools, for example, in antisense/antigene therapy. Interstrand crosslink formation in nucleic acids is one of the strategies for preparing a stable duplex by covalent bond formation. In this study, we have synthesized PNAs incorporating 4-amino-6-oxo-2-vinylpyrimidine (AOVP) as a crosslinking agent and evaluated their reactivities for targeting DNA and RNA.

## 1. Introduction

**Figure 1:**
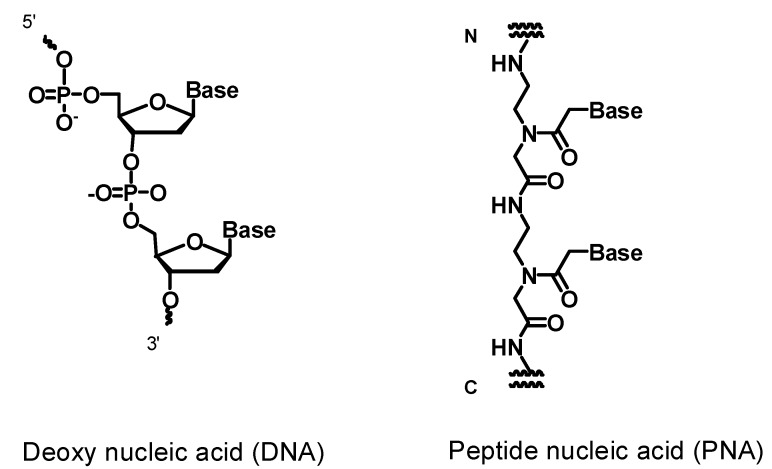
Chemical structures of DNA and PNA.

Peptide nucleic acids (PNAs) are synthetic nucleic acid analogs, in which the sugar phosphate backbone is replaced with N-(2-aminoethyl)glycine [1] (Figure 1). The hybrid complexes between PNA and DNA or RNA exhibit an extraordinary thermal stability due to the lack of a negatively-charged backbone. Additionally, PNAs are chemically stable in comparison to DNA over a wide range of temperatures and pH values and are resistant to nucleases and proteases [2]. These characteristics of the PNAs provide a variety of applications in therapeutic approaches [3,4,5], including use as biochemical tools [6]. The PNA was used as an antisense to interfere with dimerization of the HIV-1 RNA transcript and to inhibit the template switching process in HIV-1 [7]. Many modifications have been made to the PNA backbone [8] and the nucleobases [9,10,11] for improving PNA’s properties, such as their cellular uptake and solubility. GPNA is a backbone-modified PNA constructed of guanidine and is cell permeable. The GPNA designed to target the epidermal growth factor receptor (EGFR) induced potent antitumor effects due to its antisense effect [12]. A higher cellular uptake of the PNA was also achieved by conjugation with cysteine and lysine at the *N*-terminal position, which exhibited the anti-miRNA effect for miR-122 [13]. The most remarkable property of PNAs is their ability to invade the secondary structure of nucleic acids, for example, the DNA duplex [14] and the G-quadruplex [15]. This invasion ability of the PNAs raises the possibility their use to control gene expression at the DNA level with high efficiency. Strand invasion requires a high stability of the PNA-DNA complex due to competition from the displaced DNA strand. One strategy for increasing the stability of the PNA-DNA complex is by forming a covalent bond between the PNA and the target DNA.

Interstrand crosslinking (ICL) in nucleic acids is one strategy for preparing a stable duplex by covalent bond formation [16]. Several crosslinking oligonucleotides (ON) have been reported to react with DNA or RNA when activated by photo-irradiation [17] or a chemical reaction [18]. The PNA containing a modified thymine derivative is reported to form PNA/DNA ICL by photolysis or under oxidative conditions [19]. The PNA conjugated with a quinone methide precursor caused an inducible alkylation of the target DNA [20]. The bis-PNA conjugated with nitrogen mustard is reported to suppress transcription in a model system by forming a strand invasion complex and covalent bond with the target duplex DNA [21]. Thus a PNA bearing an alkylating group provides an efficient strategy for gene targeting via the inhibition of transcription with high sequence selectivity.

**Figure 2:**
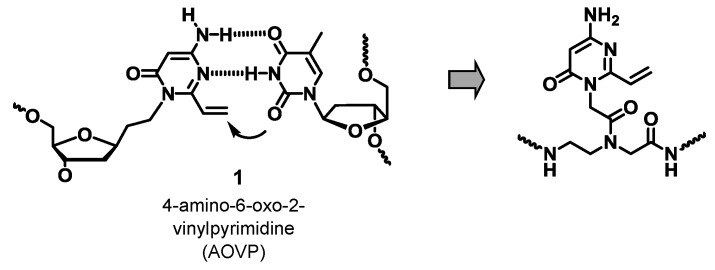
Design of the crosslink-forming PNA.

We have previously developed a 4-amino-6-oxo-2-vinyl pyrimidine (AOVP) derivative and demonstrated that this nucleoside derivative **1** exhibited a highly selective and very fast ICL reaction with thymine [22] (Figure 2). The high selectivity and reactivity of **1** could be attributed to the close proximity effect between the vinyl group of AOVP and thymine in the hybridized complex. In this paper, we describe the synthesis of PNAs containing AOVP and the evaluation of their crosslinking reactivities.

## 2. Results and Discussion

### 2.1. Synthesis of PNAs Containing 4-Amino-6-oxo-2-vinyl Pyrimidine (AOVP)

**Scheme 1:**
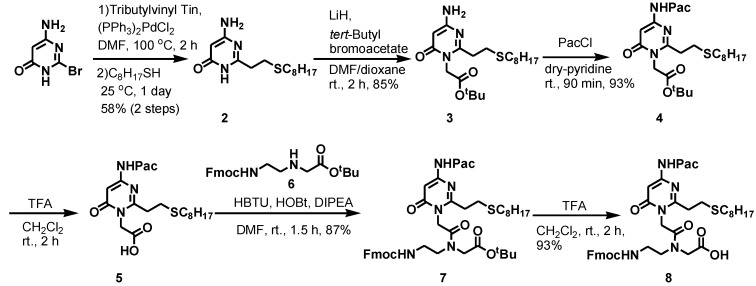
Synthesis of the AOVP PNA monomer.

The synthesis of the PNA monomer (**8**) as a stable precursor of AOVP is summarized in Scheme 1. The base portion (**2**) was synthesized as previously described [22].

The N1 position of **2** was alkylated with *t*-butyl bromoacetate to yield the *t*-butyl ester **3** in 85% yield. The 4-amino group of **3** was protected with a phenoxyacetyl group, and subsequent deprotection of the *t*-butyl group with trifluoroacetic acid (TFA) afforded the acid 5, which was coupled with the Fmoc/*t*-Bu PNA backbone **6** using HBTU/HOBT/DIPEA to form the monomer **7**, which was then deprotected of the *t*-butyl ester by treatment with TFA to produce the PNA monomer **8**. 

We designed the PNA sequence in order to target the template RNA included in the telomerase ribonucleoprotein based on a previous report, in which a quinone methide cross-linking agent was successfully incorporated [20]. It was also reported that non-covalent binding of the PNA to the template RNA of the telomerase efficiently inhibited the telomerase activity in cell cultures [23]. Therefore, we expected that our crosslinking agent in PNA would further improve the inhibitory activity for the telomerase. In this study, we synthesized two sequences that contained AOVP in the middle position or in the N-terminal position.

**Scheme 2:**
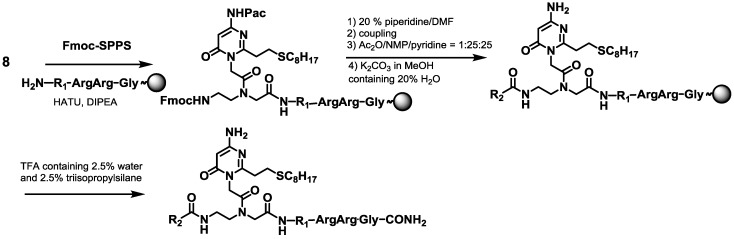
Synthesis of the PNAs containing the stable precursors of AOVP.

Generally, the C- or N-terminal position of the PNA is conjugated with lysine (Lys) to increase its solubility in water. Our previous study showed that the vinyl group of AOVP in the PNA caused an intramolecular reaction with the amino group of Lys conjugated at the C terminal position. In this study, glycine (Gly) and two arginines (Arg) were conjugated with the PNA at the C-terminal position. The solid-phase synthesis of the PNA oligomer was manually performed on the NovaSin TGR resin using Fmoc strategies. The N-terminal position of the PNA probe was modified with an acetyl group to avoid side reactions between the vinyl group in AOVP and the amino group at the N-terminal position. The resin was treated with potassium carbonate (K_2_CO_3_) in MeOH for the deprotection of the phenoxy acetyl group. Each synthesized PNA was cleaved from the resin using water/triisopropylsilane/TFA (2.5%/2.5%/95%) and purified by reverse phase HPLC to give the desired PNA**1** (Scheme 2).

**Figure 3:**
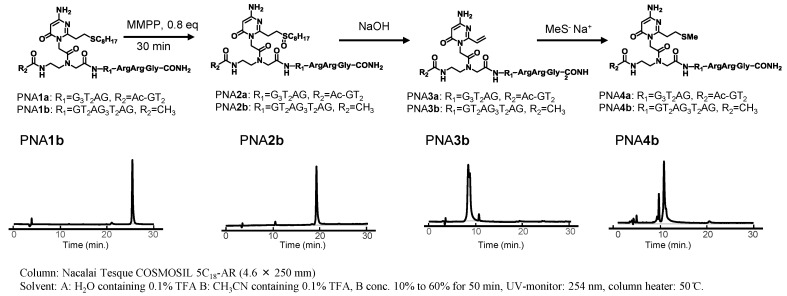
Synthesis of the PNAs containing AOVP and analysis of these reactions by HPLC.

The sulfide-protected PNA**1** was smoothly converted to PNA**2** by oxidation with magnesium monoperoxyphthalate (MMPP), followed by treatment with an aqueous NaOH solution to generate the vinyl group of PNA**3**, as shown in Figure 3. 

The structure of PNA**3** was confirmed by MALDI-TOF mass spectra measurements. The presence of the vinyl group in PNA**3** was further supported by the fact that the treatment of PNA**3** with an aqueous NaSMe solution generated the sulfide derivatives PNA**4**. 

### 2.2. Evaluation of the Crosslinking Reactivity of the PNA Containing AOVP

The crosslinking reaction was investigated under neutral conditions using the reactive PNA**3** and the target DNA (Y = dG, dA, dC, dT) or RNA (Y = rG, rA, rC, U) labelled with fluorescein at the 5' end.

**Figure 4:**
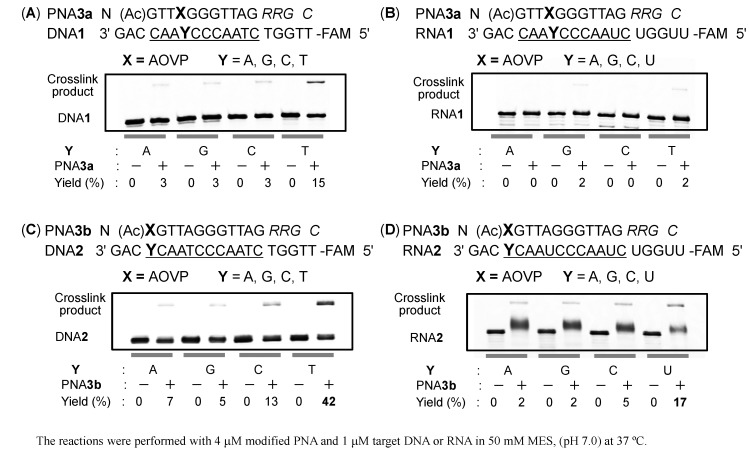
Evaluation of the crosslink reactivity of PNA**3** to DNA and RNA. (**A**) PNA3a and target DNA1; (**B**) PNA3a and target RNA1; (**C**) PNA3b and target DNA2; (**D**) PNA3b and target RNA2.

After 24 h, the reaction mixtures were analyzed by gel electrophoresis with 20% denaturing gel. The crosslinked products were identified as the less mobile bands (Figure 4). The crosslink yields were calculated from the ratio of the crosslinked product to the total amount of the remaining single stranded DNA**1**/RNA**1** and crosslinked product. PNA**3a**, which contained AOVP in the middle position, did not produced significant adducts to any targets (Figure 4A,B). Conversely the crosslink product was observed in the reaction between PNA**3b** containing AOVP at the terminal position and DNA**2** (Y=T) (Figure 4C). The crosslinking reaction of PNA**3b** did not efficiently occur with an RNA target (Figure 4D). The lower mobility bands in the reaction between PNA**3b** and DNA**2** (Y=T) was extracted from the denaturing gel and proved to be the crosslinked adduct by MALDI-TOF MS measurements (calcd.10,071.8 found 10,072.3). 

**Figure 5:**
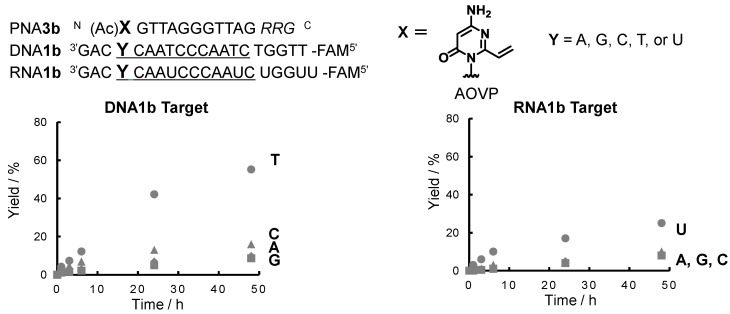
Time course of the crosslink yields with PNA**3b**.

The time course of the crosslink yields for DNA**2** and RNA**2** with PNA**3b** is summarized in Figure 5. PNA**3b** exhibited the highest yield to thymine compared to the other target bases in DNA. In contrast, lower yields with any of the target bases in RNA were observed, although the selectivity was retained to some extent during the reaction with uridine. The crosslink reactions with DNA and RNA with PNA**3b** were accelerated at 50 °C (Figure S6). The DNA oligonucleotide with the same sequence containing the AOVP derivative (**1**) did not form the duplex at 50 °C, resulting in no crosslink formation with the target DNA or RNA at this temperature. In our previous report, the AOVP derivative (**1**) at the middle position in DNA exhibited high reactivity and selectivity to thymine in DNA (~70% after 2 h) and to uridine in RNA (~80% after 2 h) [22]. The PNA CONTAINING AOVP showed lower yields than that of the DNA derivative.

**Table 1:** Melting temperatures of PNA4 with DNA and RNA.

PNA4a			PNA4b		
Target	Tm (°C)	∆Tm/°C	Target	Tm (°C)	∆Tm/°C
DNA**1** (**Y**=G)	69.7	−7.6	DNA**2** (**Y**=G)	79.2	−1.1
DNA**1** (**Y**=A)	68.0	−9.3	DNA**2** (**Y**=A)	79.2	−1.1
DNA**1** (**Y**=C)	66.1	−11.2	DNA**2** (**Y**=C)	78.6	−1.7
DNA**1** (**Y**=T)	68.1	−9.2	DNA**2** (**Y**=T)	77.9	−2.4
RNA**1** (**Y**=G)	71.3	−11.7	RNA**2** (**Y**=G)	83.2	−1.3
RNA**1** (**Y**=A)	72.9	−10.1	RNA**2** (**Y**=A)	84.1	−0.4
RNA**1** (**Y**=C)	69.1	−13.9	RNA**2** (**Y**=C)	83.2	−1.3
RNA**1** (**Y**=U)	69.1	−13.9	RNA**2** (**Y**=U)	82.9	−1.6

UV melting profiles measured using 1.5 μM each of the strands in 50 mM MES buffer, pH 7.0; PNA**5a**: GTTAGGGTTAG-RRG, PNA**5b** AGTTAGGGTTAG-RRG; Tm (°C) for PNA**5a**/DNA**1** (**Y**=T): 77.3, PNA**5a**/RNA**1** (**Y**=U): 83.0, PNA**5b**/DNA**2** (**Y**=T): 80.3, PNA**5a**/DNA**2** (**Y**=U): 84.5.

To explain the difference in the crosslink yields, the thermal stability was estimated by the melting temperature (Tm) of the duplex between PNA**4** containing the stable precursor of AOVP and the target DNA or RNA. The Tm values for PNA**4a/**DNA**1** or RNA**1** were 8–14 °C lower than those of PNA**5** containing adenosine instead of AOVP (Table 1). Conversely, no significant difference in the Tm values was observed between PNA**4b**/DNA, RNA and PNA**5b/**DNA, RNA. These results indicated that the incorporation of AOVP in the middle site may destabilize the duplex and that the incorporation of AOVP at the terminal position does not affect the thermal stability of the PNA/DNA, RNA hetero-duplex. It may be assumed that the lower reactivity of PNA**3a** might be attributed to the lower stability of the duplex. However, despite a similar stability of the duplexes formed with PNA**3b** or RNA, the reactivity of PNA**3b** with DNA was higher than that with RNA. Accordingly, the lower thermal stability of the duplex might not be the major cause of the lower reactivity of PNA containing AOVP. As the crosslink reactivity of AOVP is highly dependent on the close proximity between the reactants, a small difference in the local structure around AOVP and the target base might become a major factor of the reactivity (Figure S7). Further study about the local structure of the PNA containing AOVP is also needed for a rational explanation of the higher reactivity of PNA**3b** with DNA than that with RNA. 

## 3. Experimental Section 

### 3.1. General

All air-sensitive reactions were carried out under argon in oven-dried glassware using standard syringe and septa techniques, unless otherwise noted. The ^1^H- and ^13^C-NMR spectra (500 MHz for ^1^H and 125 MHz for ^13^C) were recorded on a Bruker spectrometer (Bruker BioSpin K.K., Kanagawa, Japan). Chemical shifts (δ) are reported in parts per million (ppm) and are referenced to the solvent CDCl_3_ (7.26 ppm), DMSO (2.50 ppm), for ^1^H-NMR and CDCl_3_ (77.16 ppm), DMSO (39.52 ppm) for ^13^C-NMR. Multiplicity and qualifier abbreviations are as follows: s = singlet, d = doublet, t = triplet, q = quartet, quint. = quintet, m = multiplet, br = broad. Coupling constants (*J*) are reported in hertz (Hz). High resolution mass spectra were obtained using electrospray ionization (ESI). MALDI-TOF mass spectra were measured by using an Autoflex Speed mass spectrometer (Bruker Daltonics, Kanagawa, Japan) with the laser at 337 nm in negative or positive mode using 3-hydroxypicolinic acid or gentisic acid as the matrix. Thin-layer chromatography was performed on Merck 60 F254 pre-coated silica gel plates (EMD Millipore Co., Darmstadt, Germany). Merck 60 F254 pre-coated silica gel on glass in a thickness of 0.9 mm was used for preparative TLC. Column chromatography was performed on silica gel (Silica Gel 60 N; 40–100 µm, or 100–210 µm, Kanto Chemical Co., Inc, Tokyo, Japan). The ultraviolet-visible (UV-vis) absorption spectra were recorded on a Beckman Coulter DU800 (Beckman Coulter K.K., Tokyo, Japan). PNA synthesis was carried out by Fmoc-SPPS. High performance liquid chromatography (HPLC) was performed using Nacalai Tesque (Kyoto, Japan) Cosmosil 5C18AR (4.6 or 10 × 250 mm) as the column, a JASCO PU-2089 as the pump, JASCO 2075 for the UV monitoring, and JASCO 2067 as the column oven (all Jasco Co., Tokyo, Japan). pH measurements were measured performed with a Mettler Toledo (Schwerzenbaha, switzerland) Seven Easy pH meter using a 8220BNWP electrode (Thermo Scientific, Waltham, MA, USA). UV-melting experiments were performed on a Beckman Coulter DU800. Densitometric analysis of the gel was carried out on 20% denaturing polyacrylamide gel plates, and visualized and quantified using a FLA-5100 Fluor Imager (Fujifilm Co., Tokyo, Japan). Commercially available reagents were obtained from Wako Pure Chemical Industries Ltd. (Osaka, Japan) or Kanto Chemical Co., Inc. and used without further purification. DNA and RNA oligomers were purchased from Japan Bio Services Co., Ltd. (Saitama, Japan), buffers and salts were from Nacalai Tesque. 

### 3.2. Chemistry

#### 3.2.1. Synthesis of 1-(*tert*-Butoxycarbonylmethyl)-4-amino-6-oxo-2-(2-octylthioethyl)pyrimidine (**3**) 

Compound **2** (300 mg, 1.1 mmol) and lithium hydride (84 mg, 11 mmol) were dissolved in dry dioxane (20 mL) and dry DMF (10 mL), and the reaction mixture was stirred at room temperature. After 1.5 h, *tert*-butyl bromoacetate (233 μL, 1.6 mmol) was added and stirred at room temperature. After 1.5 h, the reaction mixture was diluted with 50 mL EtOAc and washed with sat. NH_4_Cl aq. (50 mL), and brine (50 mL). The organic layer was dried over Na_2_SO_4_, filtered, and concentrated under reduced pressure. The residue was purified by column chromatography (hexane/EtOAc = 1:1) to give **3** as a colorless oil (362 mg, 0.91 mmol 87%). ^1^H-NMR (CDCl_3_): δ 5.35 (s, 1H), 4.69 (s, 2H), 4.67 (s, 2H), 2.92–2.89 (m, 2H), 2.80–2.76 (m, 2H), 2.53 (t, *J* = 7.5 Hz, 2H), 1.59 (quin, *J* = 7.5Hz, 2H), 1.48 (s, 9H), 1.38–1.27 (m, 10H), 0.88 (t, *J* = 7.5 Hz, 3H). ^13^C-NMR (CDCl_3_): δ 167.1, 162.8, 161.3, 160.3, 85.5, 83.1, 44.5, 35.2, 32.7, 31.9, 29.8, 29.3, 29.0, 28.5, 28.1, 22.7, 14.2. HRMS (ESI-MS) *m/z* calcd for C_20_H_35_N_3_O_3_S [M+H]^+^ 398.24719, found 398.24711.

#### 3.2.2. Synthesis of 1-(*tert*-Butoxycarbonylmethyl)-4-phenoxyacetylamino-6-oxo-2-(2-octylthioethyl)pyrimidine (**4**)

To a solution of **3** (130 mg, 0.33 mmol) in dry pyridine (5 mL) was added phenoxyacetyl chloride (68 µL, 4.9 mmol), and the reaction mixture was stirred at room temperature. After 1.5 h, the reaction mixture was diluted with 50 mL EtOAc and washed with water (50 mL), and brine (50 mL). The organic layer was dried over Na_2_SO_4_, filtered, and concentrated under reduced pressure. The residue was purified by column chromatography (CH_2_Cl_2_/EtOAc=9:1 to 4:1) to give **4** as a white solid (169 mg, 0.32 mmol, 93%). ^1^H-NMR (CDCl_3_): δ 8.49 (s, 1H), 7.37–7.33 (m, 2H), 7.25 (s, 1H), 7.08–7.05 (m, 1H), 7.00–6.98 (m, 2H), 4.70 (s, 2H), 4.59 (s, 2H), 2.93–2.90 (m, 2H), 2.85–2.82(m, 2H), 2.54 (t, *J* = 7.5 Hz, 2H), 1.59 (quin, *J* = 7.5Hz, 2H), 1.48 (s, 9H), 1.39–1.26 (m, 10H), 0.87 (t, *J* = 7.0 Hz, 3H). ^13^C-NMR (CDCl_3_): δ 167.3, 166.4, 162.9, 160.5, 157.0, 152.8, 130.1, 122.8, 115.1, 97.0, 83.5, 67.7, 45.0, 35.1, 32.8, 31.9, 29.8, 29.3, 29.0, 28.5, 28.1, 22.7, 14.2. HRMS (ESI-MS) *m/z* calcd for C_28_H_41_N_3_O_5_S [M+H]^+^ 532.28397, found 532.28398.

#### 3.2.3. Synthesis of 1-(Carbonylmethyl)-4-phenoxyacetylamino-6-oxo-2-(2-octylthioethyl)pyrimidine (**5**)

To a solution of **4** (200 mg, 0.38 mmol) in CH_2_Cl_2_ (1 mL) was added TFA (9 mL) and the reaction mixture was stirred for 1 h at room temperature. The solvent was removed under reduced pressure and the residue was diluted with 5 mL diethyl ether. The solution was added to hexane (30 mL) to cause precipitation. The resulting precipitates were collected by filtration and washed with hexane/diethyl ether (9:1) to give a pure sample of **5** as a white powder (131 mg, 0.28 mmol, 89%). ^1^H-NMR (DMSO): δ 10.4 (s, 1H), 7.30 (t, *J* = 8.0 Hz, 2H), 6.98–6.92 (m, 3H), 6.86 (s, 1H), 4.81 (s, 2H), 4.75 (s, 2H), 2.96 (t, *J* = 7.0 Hz, 2H), 2.88 (t, *J* = 7.0 Hz, 2H), 2.54 (t, *J* = 7.5 Hz, 2H), 1.52 (quin, *J* = 7.5 Hz, 2H), 1.34–1.24 (m, 10H), 0.85 (t, *J* = 7.0 Hz, 3H). ^13^C-NMR (DMSO): δ 169.1, 168.3, 162.1, 161.0, 157.7, 153.8, 129.5, 121.1, 114.4, 94.7, 66.6, 44.3, 34.2, 31.2, 31.1, 29.0, 28.6, 28.5, 28.2, 27.3, 22.0, 13.9. HRMS (ESI-MS) *m/z* calcd for C_24_H_33_N_3_O_5_S [M+H]^+^ 476.22137, found 476.22136.

#### 3.2.4. Synthesis of *tert*-Butyl-*N*-[2-(*N*-9-fluorenylmethoxycarbonyl)aminoethyl]-*N*-[[4-phenoxyacetyl-amino-6-oxo-2-(2-octylthioethyl)pyrimidin-1-yl]acetyl]glycinate (**7**)

Compound **5** (100 mg, 0.21 mmol) was dissolved in dry DMF (5 mL). HBTU (159 mg, 0.42 mmol), HOBt (57 mg, 0.42 mmol), and DIPEA (146 µL, 0.84 mmol) were added and stirred for 5 min at room temperature. The reaction mixture was added to a solution of **6** (82 mg, 0.21 mmol) in dry DMF (5 mL) and stirred at room temperature. After 1.5 h, the reaction mixture was diluted with 50 mL EtOAc, and washed with sat.NaHCO_3_ (2 × 50 mL) and brine (50 mL). The organic layer was dried over Na_2_SO_4_, filtered, and concentrated under reduced pressure. The residue was purified by column chromatography (hexane/EtOAc=2:1) to give **7** as a white foam (140 mg, 0.16 mmol, 87%). ^1^H-NMR (DMSO): δ 10.42 (s, 1H), 7.81 (d, *J* = 7.5 Hz, 2H), 7.68 (t, *J* = 7.0 Hz, 2H), 7.44–7.39 (m, 2H), 7.33–7.29 (m, 4H), 6.96–6.92 (m, 3H), 6.85 (s, 1H), 5.07 and 4.86 (br s, 2H), 4.81 (s, 2H), 4.35–4.20 (m, 4H), 3.95 (s, 1H), 3.51–3.12 (m, 4H), 2.86 (br s, 4H), 1.48–1.39 (m, 11H), 1.29–1.20 (m, 10H), 0.84–0.82 (m, 3H). ^13^C-NMR (DMSO): δ 168.6, 168.3, 168.2, 167.9, 166.9, 166.6, 162.0, 161.50, 161.45, 157.7, 156.4, 156.1, 153.7, 143.8, 142.6, 140.7, 139.4, 137.4, 129.5, 128.9, 127.6, 127.2, 127.0, 125.1, 121.3, 121.1, 120.1, 120.0, 114.4, 109.6, 94.6, 82.0, 80.9, 66.6, 65.6, 65.4, 50.0, 49.0, 47.3, 46.7, 43.7, 43.3, 38.0, 34.0, 31.2, 31.09, 31.05, 29.0, 28.6, 28.5, 28.24, 28.21, 27.6, 27.4, 27.3, 22.0, 13.9. HRMS (ESI-MS) *m/z* calcd for C_47_H_59_N_5_O_8_S [M+Na]^+^ 876.39766, found 876.39760.

#### 3.2.5. Synthesis of *N*-[2-(*N*-9-Fluorenylmethoxycarbonyl)aminoethyl]-*N*-[[4-phenoxyacetyl-amino-6-oxo-2-(2-octylthioethyl)pyrimidin-1-yl]acetyl]glycine (**8**)

To a solution of **7** (40 mg, 47 µmol) in CH_2_Cl_2_ (1 mL) was added TFA (9 mL) and the resulting mixture was stirred at room temperature for 1.5 h. The solvent was removed and the residue was diluted with 5 mL diethyl ether. The solution was added to hexane (30 mL) to cause the precipitation. The resulting precipitates were collected by filtration and washed with hexane/diethyl ether (9/:1) to give a pure sample of **8** as a white powder (34 mg, 41 µmol, 93%). ^1^H-NMR (DMSO): δ 10.42 and 10.41 (s, 1H), 7.88 (d, *J* = 7.5 Hz, 2H), 7.68 (t, *J* = 7.5 Hz, 2H), 7.46–7.39 (m, 2H), 7.33–7.39 (m, 4H), 6.98–6.92 (m, 3 H), 6.85 (s, 1H), 5.06 and 4.89 (br s, 2H), 4.81 (s, 2H), 4.34–4.21 (m, 4H), 4.00 (s, 1H), 3.52–3.13 (m, 4H), 2.85 (br s, 4H), 1.46–1.45 (m, 2H), 1.27–1.19 (m, 10H), 0.84–0.80 (m, 3H). ^13^C-NMR (DMSO): δ 170.9, 170.2, 168.3, 167.0, 166.6, 162.0, 161.6, 161.5, 157.7, 156.4, 156.1, 153.7, 143.9, 140.7, 129.5, 127.6, 127.0, 125.1, 121.1, 120.1, 114.4, 94.6, 66.6, 65.6, 65.5, 49.2, 47.9, 47.2, 46.7, 43.7, 43.4, 38.0, 34.04, 33.96, 31.2, 31.12, 31.06, 29.0, 28.60, 28.57, 28.2, 27.44, 27.35, 22.0, 13.9. HRMS (ESI-MS) *m/z* calcd for C_43_H_51_N_5_O_8_S [M+Na]^+^ 820.33506, found 820.33508.

#### 3.2.6. Synthesis of PNA**1**

Modified PNAs were synthesized on Novasyn TGR resin by the Fmoc-SPPS procedure. After acetylation of the N-terminal amino group, the resin was treated with 0.1 M K_2_CO_3_ in MeOH containing 20% H_2_O for 3 h for deprotection of the Pac group in the precursor of AOVP. The resulting oligomer was cleaved from the resin by treatment with a cleaving cocktail containing water/triisopropylsilane/TFA (12.5:12.5:475 μL for 20 mg of resin) for 90 min. The crude mixture was eluted and precipitated in diethyl ether, dissolved in water containing 0.1% TFA, purified by reversed-phase HPLC, and characterized by MALDI-TOF mass spectrometry. The concentration of each oligomer was determined by UV absorption at 260 nm in water using the following extinction coefficients: 115,800 M^−1^ cm^−1^ (PNA**1a**), 131,000 M^−1^ cm^−1^ (PNA**1b**). MALDI-TOF MS (*m*/*z*) PNA**1a**: calcd 3647.5, found 3,647.4, PNA**1b**: calcd 3922.7, found 3922.3.

#### 3.2.7. Synthesis of PNA**3**

To a solution of PNA**1** (0.1 mM, 5 μL, 0.5 nmol) was added a solution of magnesium monoperoxyphthalate (MMPP) (0.5 mM, 0.6 μL, 0.3 nmol) in carbonate buffer adjusted to pH 10 at room temperature. After 30 min, a solution of NaOH (0.1 M, 0.5 μL, 50 nmol) was added, and the mixture was left for an additional 1 h to give PNA**3**. MALDI-TOF MS (*m*/*z*) PNA**3a**: calcd 3500.4, found 3500.4, PNA**3b**: calcd 3776.6, found 3776.0.

#### 3.2.8. Synthesis of PNA**4**

A solution of sodium thiomethoxide (1 M, 0.5 μL, 0.5 μmol) was added to the above mentioned mixture of PNA**3**. The mixture was left for 1h and purified by HPLC to give PNA**4**. MALDI-TOF MS (*m*/*z*) PNA**4a**: calcd 3549.4, found 3549.9, PNA**4b**: calcd 3824.6, found 3824.5.

### 3.3. General Procedure for the Crosslinking Reaction 

The crosslinking reaction was performed with 4 μΜ of **PNA3** and 1 μΜ of the target DNA or RNA labeled by fluorescein at the 5'-end in a buffer of 50 mM MES (pH 7.0). The reaction mixture was incubated at 37 °C. An aliquot of the reaction mixture was collected at each indicated time and quenched by the addition of loading dye (formamide [~100% v/v], 0.5 M EDTA [20 µL: 1 mM], xylene cyanol [0.05% w/v], and bromophenol blue [0.05% w/v]). The cross-linked products were analyzed by denaturing 20% polyacrylamide gel electrophoresis containing urea (7 M) with TBE buffer [PNA**3a**] or denaturing 20% polyacrylamide gel electrophoresis containing urea (7 M) and 10% v/v formamide at 50 °C with TBE buffer [PNA**3b**]. The labeled bands were visualized and quantified using a FLA-5100 Fluor Imager.

### 3.4. Melting Temperature (T_m_) Measurements

UV-melting experiments were performed on a Beckman Coulter DU800. For the hetero-duplex formation study, equimolar amounts of each oligonucleotide and PNA were dissolved in 50 mM MES buffer (pH 7.0) to provide final concentrations of 1.5 µM. The solutions were heated to 90 °C for 5 min and allowed to cool to room temperature slowly. The melting profiles were recorded at 260 nm from 20 to 90 °C at a scan rate of 1 °C/min.

## 4. Conclusions 

In this study, we have synthesized PNAs containing AOVP as a crosslinking agent and evaluated their reactivities toward the targets DNA and RNA. The PNA incorporating AOVP at the terminal position exhibited a highly selective crosslinking reactivity to the thymine in DNA and lower reactivity to any bases in RNA. This PNA is expected to provide a new probe for sequence specific PNA-DNA interactions for a regulation of the gene expression. The application of the PNA-incorporated AOVP for crosslinking reactions with the duplex DNA by forming an invasion complex is currently under investigation.

## Supplementary Materials

Supplementary materials can be accessed at: http://www.mdpi.com/1420-3049/20/03/4708/s1.
