# Supplementary material for: Synthesis of Peptide Nucleic Acids Containing a Crosslinking Agent and Evaluation of Their Reactivities
Source: Molecules. 2015 Mar 13;20(3):4708–19. doi: 10.3390/molecules20034708 (PMC6272193; doi:10.3390/molecules20034708)
Supplement: Supplementary file 1 [file molecules-20-04708-s001.pdf]

Supporting Information

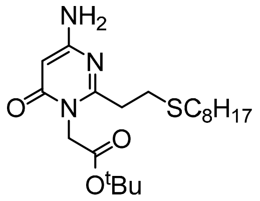

3

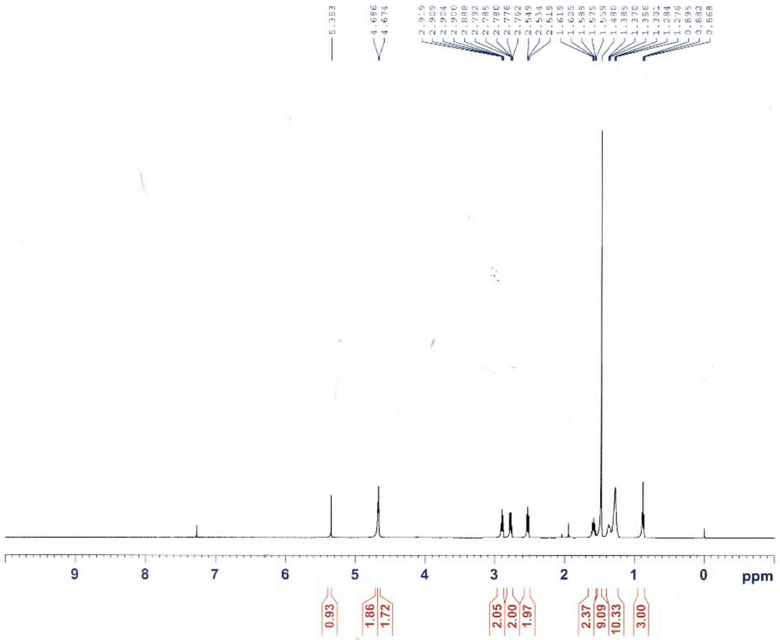

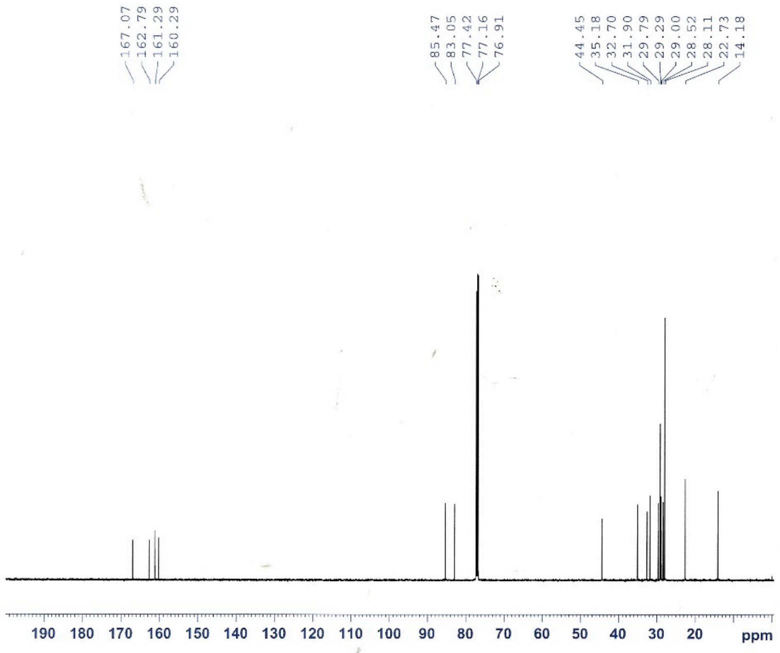

Figure S1. ^{1}H-NMR and ^{13}C-NMR spectra of **3**.

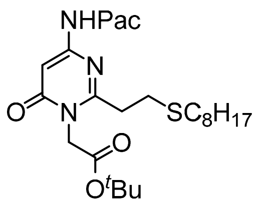

4

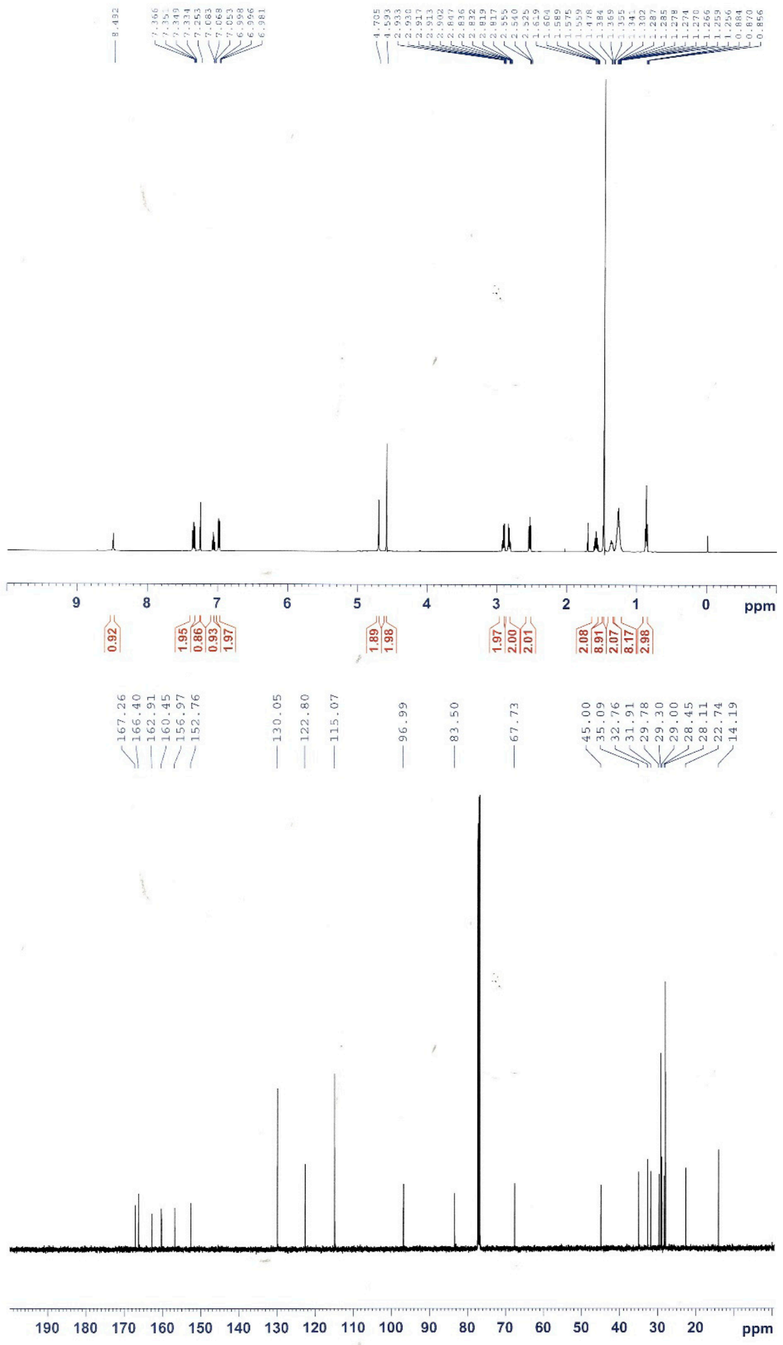

Figure S2. ^{1}H-NMR and ^{13}C-NMR spectra of **4**.

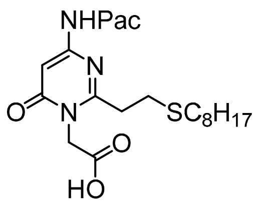

5

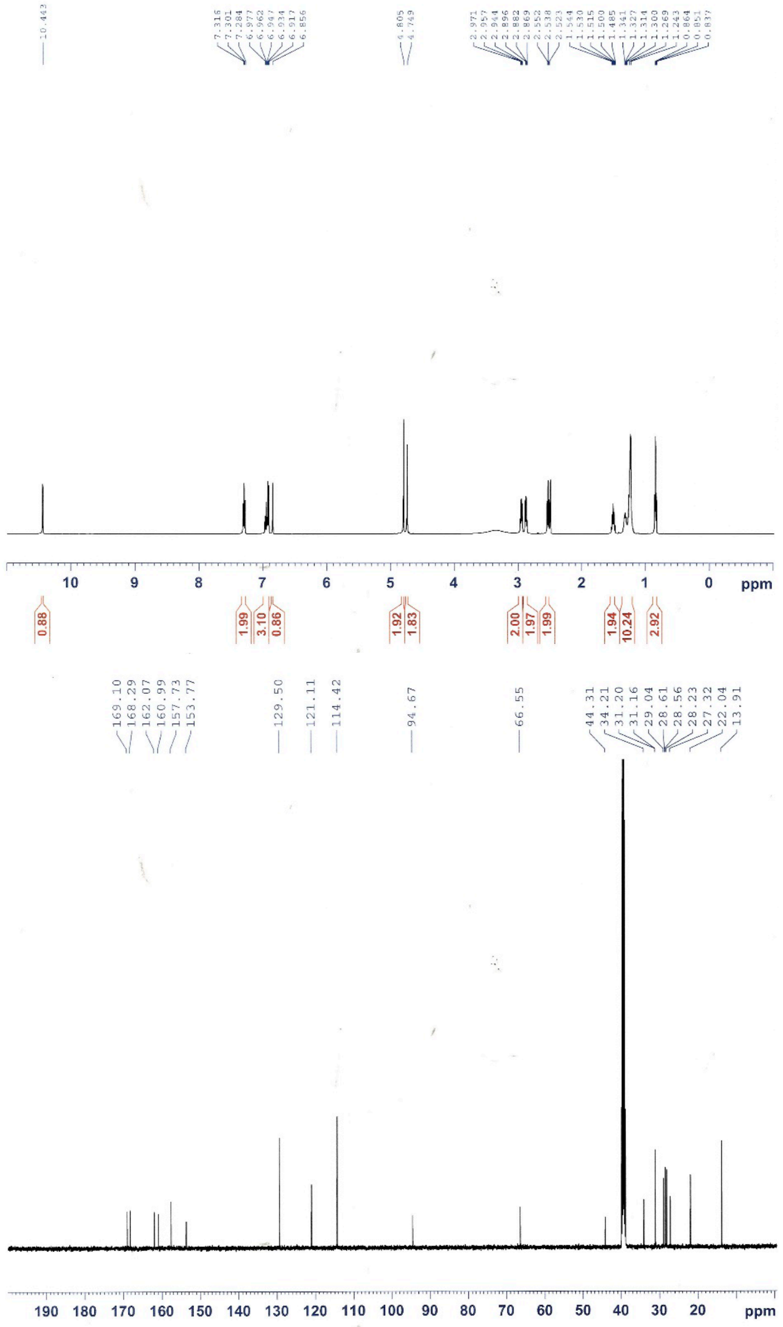

Figure S3. ^{1}H-NMR and ^{13}C-NMR spectra of **5**.

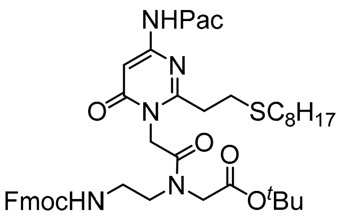

7

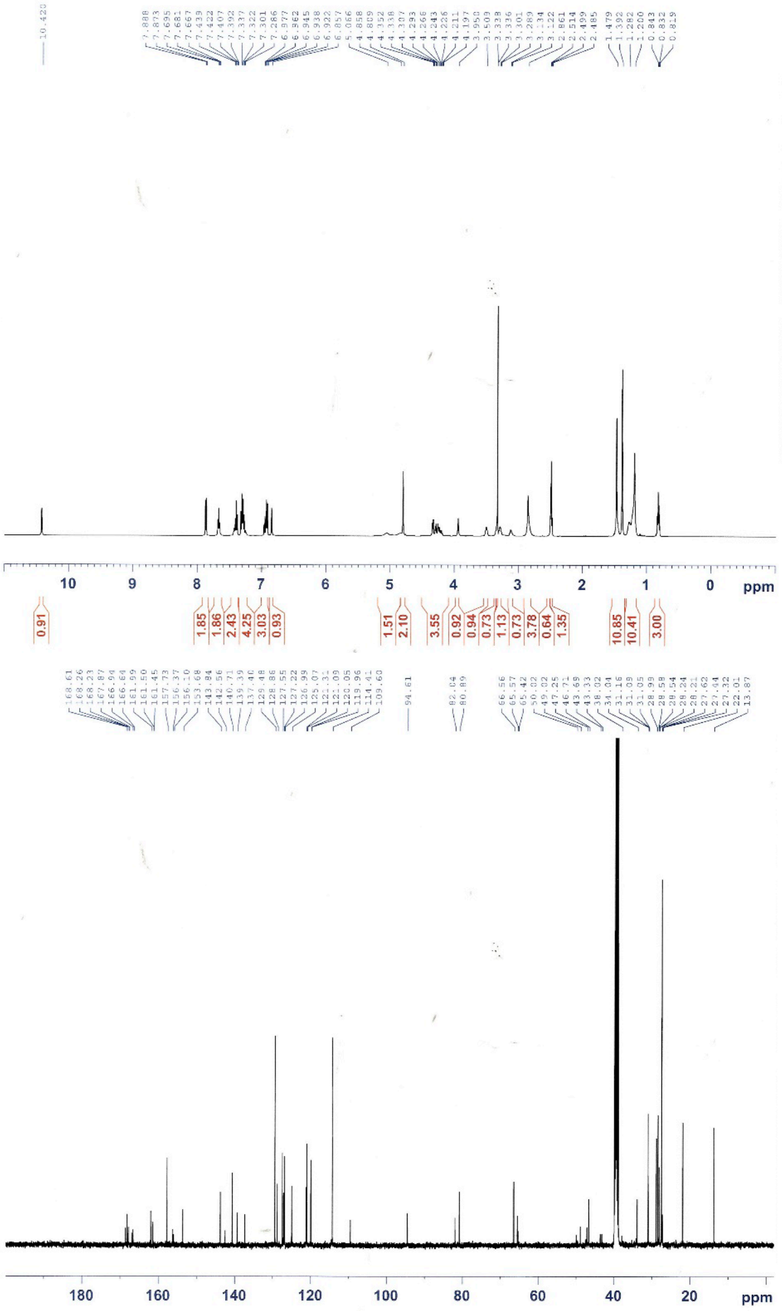

Figure S4. ^{1}H-NMR and ^{13}C-NMR spectra of **7**.

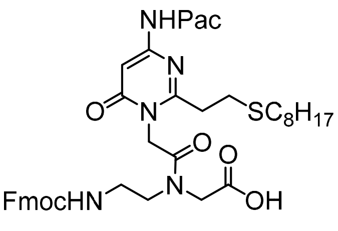

8

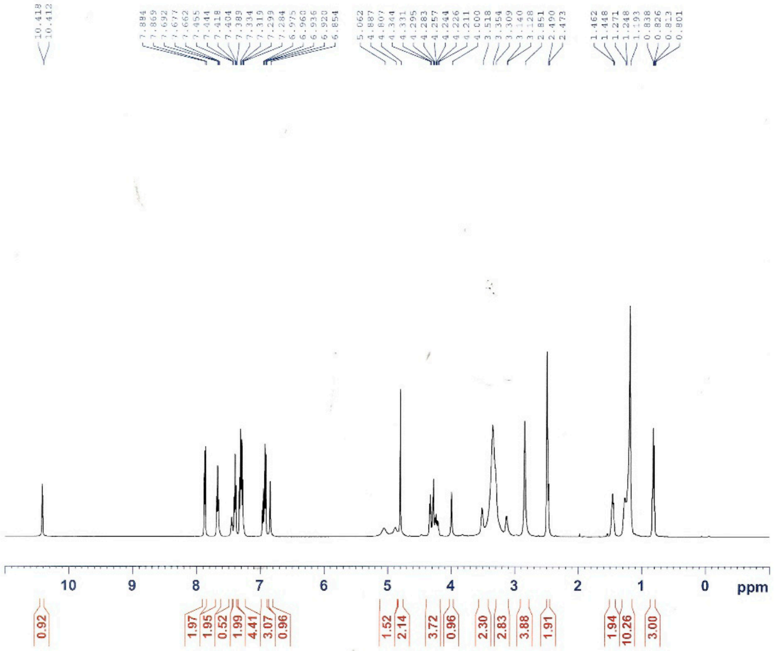

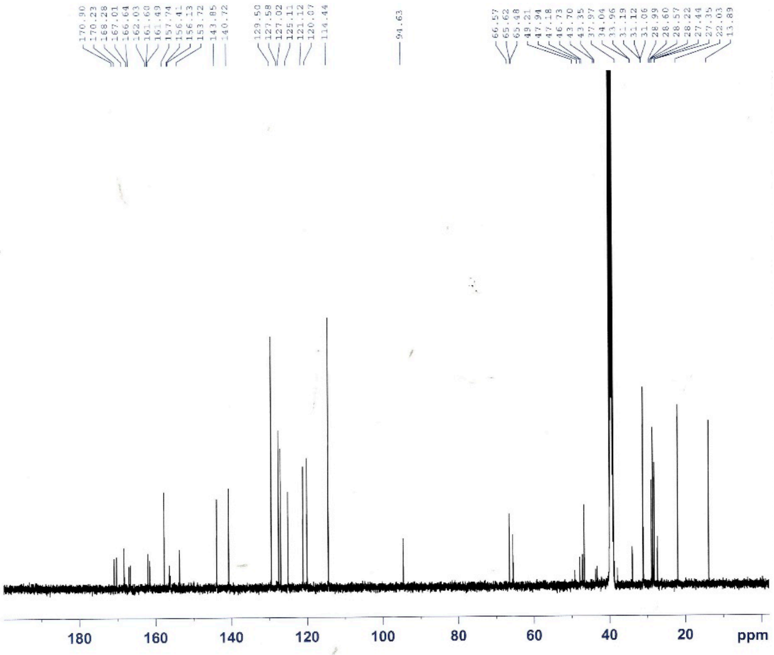

Figure S5. ^{1}H-NMR and ^{13}C-NMR spectra of **8**.

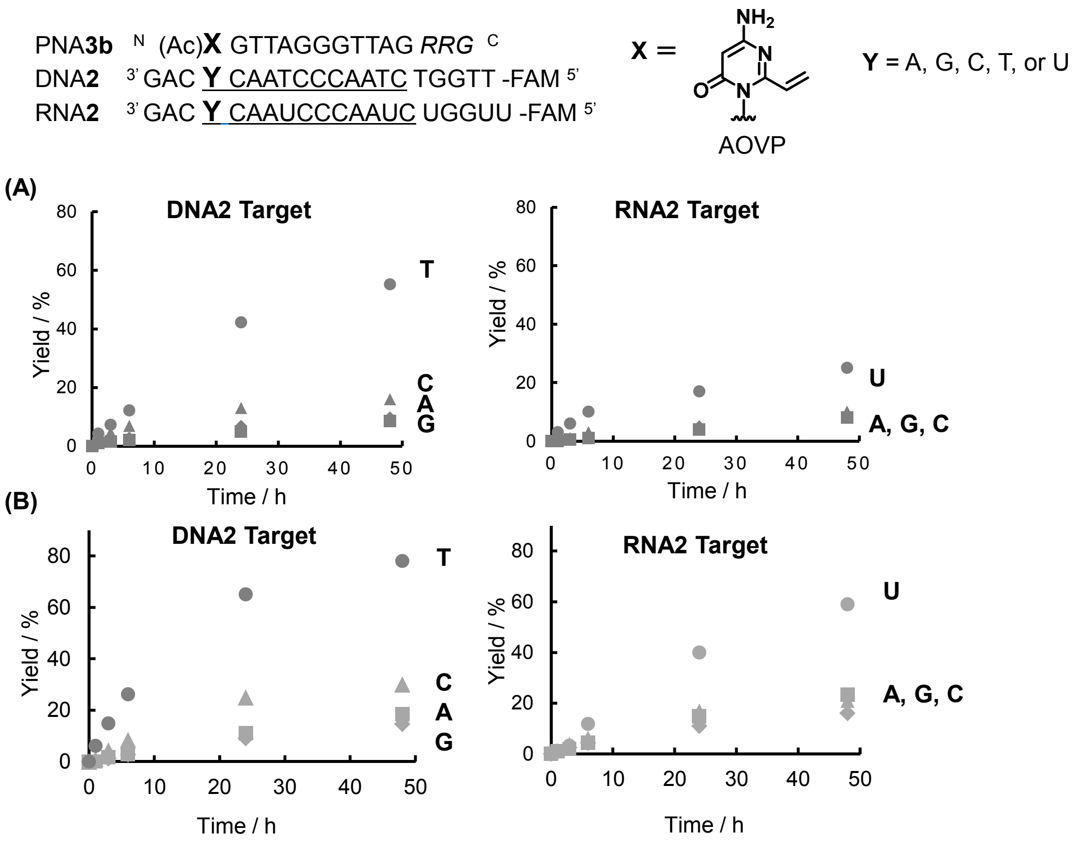

Figure S6. Time course of the crosslink yields with PNA**3b** at 37 °C (**A**) and 50 °C (**B**).

(A)

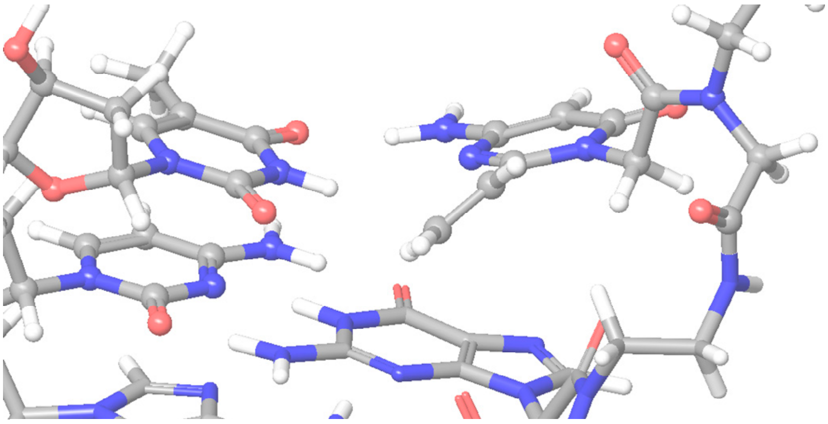

(B)

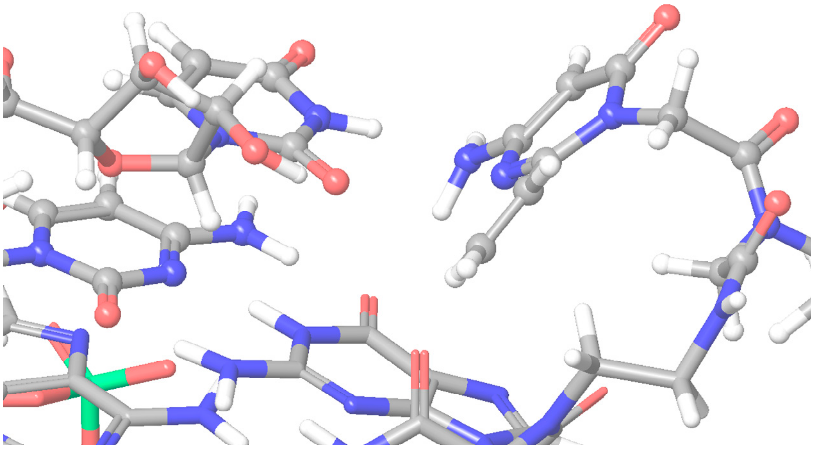

Figure S7. Molecular modeling of the complex in the PNA**3b**/DNA (**A**) and PNA**3b**/RNA (**B**). Molecular modeling of the complex was performed with MacroMoldel using OPLS2005 in water. These results suggested that AOVP in PNA might form the two hydrogen bonds with a target thymine in DNA and showed higher reactivity by the proximity effect. On the other hand, AOVP in PNA might not form the hydrogen bond with a target uracil in RNA. Thus, PNA**3b** might exhibit the higher reactivity with DNA than RNA.
